# Editorial: Endocrine Forms of Hypertension: Clinical and Emerging Molecular Aspects

**DOI:** 10.3389/fendo.2019.00857

**Published:** 2019-12-06

**Authors:** Peter Igaz, Teresa Maria Seccia

**Affiliations:** ^1^Second Department of Internal Medicine, Faculty of Medicine, Semmelweis University, Budapest, Hungary; ^2^MTA-SE Molecular Medicine Research Group, Hungarian Academy of Sciences, Semmelweis University, Budapest, Hungary; ^3^Clinica dell’Ipertensione Arteriosa, Department of Medicine, University of Padua, Padua, Italy

**Keywords:** hypertension, primary aldosteronism, thyroid, licorice, Cushing's syndrome, microRNA

Secondary hypertension is responsible for about 5–20% of all hypertension cases depending on the examined cohorts (1–3). In contrast with primary essential hypertension that can only be treated, secondary hypertension often manifesting itself as resistant hypertension includes potentially curable diseases (1–3). Research on endocrine diseases leading to secondary hypertension is a progressive field, and better elucidating their pathophysiology and clinical features, might pave the way for novel diagnostic modalities and treatments. The major endocrine diseases as causes of hypertension include primary aldosteronism, hypercortisolism, pheochromocytoma/paraganglioma, thyroid diseases, primary hyperparathyroidism, and acromegaly.

In this Research Topic, we have compiled several articles on different issues of endocrine secondary hypertension regarding both molecular and clinical features. Primary aldosteronism is the most common endocrine cause of secondary hypertension affecting 5–13% of hypertensive patients in different studies (4–6). Three articles are dedicated to its field in this Research Topic.

The study by Mohideen et al. was aimed at establishing the prevalence of *KCNJ5* gene mutations in APAs from a Malaysian cohort. The authors, by examining 54 APAs from a Malaysian cohort, found that its prevalence was 31%, similarly as reported in Caucasians, however, with no prevalence of mutants in females. *KCNJ5* mutant and wild type APAs showed similar percentages of ZF- and ZG- like cells, but *KCNJ5* mutant APAs with a ZF-like profile tended to be associated with larger APA. A higher expression of *CYP11B2* was found in females, who also had adrenalectomy at a younger age than males. An interesting hypothesis was provided by the Authors: since females had often larger tumors, the phenotype previously associated with *KCNJ5* mutant APAs could be the phenotype of APAs from female patients, not the phenotype of all KCNJ5 mutant APAs.

In the original study by Decmann et al., circulating microRNAs belonging to the group of non-coding RNA molecules (7, 8) are investigated as potential novel diagnostic tools for the differentiation of the two major forms of primary aldosteronism i.e., bilateral adrenal hyperplasia and unilateral adenoma. Three microRNAs were validated on a large cohort of samples to be overexpressed in bilateral hyperplasia relative to the unilateral adenoma samples. Although sensitivity and specificity values were not found to be high enough for clinical introduction at present, this study presents a novel class of potential diagnostic markers for differentiating subclasses of endocrine hypertension, but also underlines that primary aldosteronism could be regarded as a spectrum disease.

A comprehensive review on licorice-induced pseudohyperaldosteronism is given by Sabbadin et al., who discussed its biochemical picture and the mechanisms underlying the mineralocorticoid effect. Of interest, they also discuss the potential therapeutic use of licorice related to its anti-androgen and estrogen-like activity, mostly exploited for treatment of polycystic ovary syndrome on top of spironolactone, and the anti-inflammatory effects of licorice, unveiling unfamiliar properties of an old medicinal plant.

The pathophysiology and treatment of hypertension in Cushing's syndrome is discussed in the mini review by Barbot et al.. Several relevant molecular mechanisms are presented, and most notably antihypertensive and also cortisol-lowering treatments are discussed. Clinically relevant issues are also discussed in the mini review by Canu et al. where the hypertension-related clinical picture, genetics and treatment issues of pheochromocytoma are presented.

Thyroid diseases are very frequent, but they less often affect blood pressure values. A comprehensive view of the effects of thyroid hormones on the cardiovascular system is provided by Berta et al., who also discuss the genetic background that may favor cardiovascular damage.

We hope that the reader will find this Research Topic interesting, and the molecular and clinical issues covered by the articles will be helpful in both research and clinical management of secondary endocrine hypertension.

## Author Contributions

Both authors listed have made a substantial, direct and intellectual contribution to the work, and approved it for publication.

### Conflict of Interest

The authors declare that the research was conducted in the absence of any commercial or financial relationships that could be construed as a potential conflict of interest.
